# Perceptions of the elderly on ageing in place in Hong Kong

**DOI:** 10.1186/2193-1801-4-S2-O4

**Published:** 2015-11-27

**Authors:** Kris Wai-ning Wong, Stella Sin-tung Kwok, Fiona Wing-yin Luk

**Affiliations:** Li Ka Shing Institute of Professional and Continuing Education, The Open University of Hong Kong, Hong Kong; Research Support Unit, Vocational Training Council, Hong Kong

## Background

To serve the diverse needs of the ageing population, the Chief Executive of the Hong Kong Special Administrative Region has made 'Care for the Elderly' a strategic policy objective of the government since 1997. The objective of the policy is to improve the quality of life of our elderly population and provide them with a sense of security, a sense of belonging and a feeling of health and worth. 'Ageing in place' has been government policy since 2003. However, no studies have been conducted in Hong Kong to investigate how the elderly perceive the policy and practice of ageing in place. This qualitative exploratory study investigated the understanding, attitudes and concerns of elderly people in Hong Kong towards ageing in place. The results provide valuable information for the Hong Kong government to develop appropriate measures to facilitate the provision of ageing in place as the core policy, with institutional care as a back-up.

## Methods

One hundred and six participants (49 male and 57 female) aged between 65 and 78 took part in focus group interviews between August 2014 and January 2015. The participants were questioned about their perceptions on ageing in place and the essential elements that facilitate it. They were also asked about their opinions on strategic policy and practice in the provision of ageing in place. All interviews were tape-recorded and transcribed. Two independent researchers coded the transcribed interviews and conducted content analysis with the aid of NVivo 10.

## Results

The majority of participants in this study preferred to age in place because it increased their sense of belonging, security and familiarity and is more convenient. The essential elements for ageing in place included home attributes, the neighbourhood environment, community network, social security and medical support, all of which could affect the quality of life of the elderly.

## Conclusion

The results of this study provide a reference on the essential elements that should be included in developing and implementing policies on ageing in place. With appropriate infrastructure and social support, the government could facilitate the elderly to live independent lives and maximise their abilities.

